# Prolonged Exposure to Simulated Microgravity Changes Release of Small Extracellular Vesicle in Breast Cancer Cells

**DOI:** 10.3390/ijms232416095

**Published:** 2022-12-17

**Authors:** Petra M. Wise, Jayashree Sahana, Paolo Neviani, Thomas Juhl Corydon, Herbert Schulz, Markus Wehland, Manfred Infanger, Daniela Grimm

**Affiliations:** 1 The Saban Research Institute, Children’s Hospital Los Angeles, University of Southern California, 4650 Sunset Blvd, Los Angeles, CA 90027, USA; 2 Department of Microgravity and Translational Regenerative Medicine, University Clinic for Plastic, Aesthetic and Hand Surgery, Otto von Guericke University, Universitätsplatz 2, 39106 Magdeburg, Germany; 3 Research Group “Magdeburger Arbeitsgemeinschaft für Forschung unter Raumfahrt- und Schwerelosigkeitsbedingungen” (MARS), Otto von Guericke University, 39106 Magdeburg, Germany; 4 Department of Biomedicine, Aarhus University, Ole Worms Allé 4, 8000 Aarhus C, Denmark; 5 Department of Ophthalmology, Aarhus University Hospital, Palle Juul-Jensens Blvd. 99, 8200 Aarhus N, Denmark; 6 Plastic, Aesthetic and Hand Surgery, Otto-von-Guericke University Clinic, Leipziger Str. 44, 39120 Magdeburg, Germany

**Keywords:** breast cancer, extracellular vesicles, exosomes, microgravity, tetraspanins, cell-cell communication

## Abstract

Breast cancer is the leading cause of cancer incidence worldwide and among the five leading causes of cancer mortality. Despite major improvements in early detection and new treatment approaches, the need for better outcomes and quality of life for patients is still high. Extracellular vesicles play an important role in tumor biology, as they are able to transfer information between cells of different origins and locations. Their potential value as biomarkers or for targeted tumor therapy is apparent. In this study, we analyzed the supernatants of MCF-7 breast cancer cells, which were harvested following 5 or 10 days of simulated microgravity on a Random Positioning Machine (RPM). The primary results showed a substantial increase in released vesicles following incubation under simulated microgravity at both time points. The distribution of subpopulations regarding their surface protein expression is also altered; the minimal changes between the time points hint at an early adaption. This is the first step in gaining further insight into the mechanisms of tumor progression, metastasis, the education of the tumor microenvironments, and preparation of the metastatic niche. Additionally, this may lighten up the processes of the rapid cellular adaptions in the organisms of space travelers during spaceflights.

## 1. Introduction

Female breast cancer is one of the scourges of our time, becoming the most common cancer globally in 2020 by surpassing lung cancer as the leading cause of global cancer incidence. With an estimated 2.3 million new cases, representing 11.7% of all cancer cases worldwide in both sexes, and 685,000 deaths, breast cancer is the fifth leading cause of cancer mortality [1,2]. Among women, it accounts for 1 in 4 cancer cases and 1 in 6 cancer deaths, making it first in ranking for incidence globally [1]. In 2022, an estimated 287,850 new cases of invasive breast cancer are expected to be diagnosed in women in the U.S. alone, along with 51,400 new cases of non-invasive (in situ) breast cancer [2]. Causes and risk factors for the development of breast cancer are numerous and include genetic predisposition, reproductive and hormonal risk factors (early age at menarche, later age at menopause, advanced age at first birth, fewer number of children, less breastfeeding, menopausal hormone therapy, oral contraceptives) and lifestyle risk factors such as alcohol intake, excess body weight, and physical inactivity [3]. Among hereditary cases, almost a fourth result from a mutation in one of a few rare but highly penetrant genes, including breast cancer 1 (*BRCA1*), breast cancer 2 (*BRCA2*), phosphatase and tensin homolog (*PTEN*), tumor protein P53 (*TP53*), E-cadherin (*CDH1*) and serine/threonine kinase 11 (*STK11*), which present up to an 80% lifetime risk of developing breast cancer [4]. The relationship between breast cancer development and outcomes and certain lifestyle factors is an area that has been researched extensively [5].

Breast cancer is the most invasive cancer in women, and the immense tumor heterogeneity is the major issue limiting the efficacy of targeted cancer therapies. Seven subtypes of female breast cancer can be differentiated, depending on their molecular characteristics, and stratification into these subtypes, along with staging, directs the course of treatment and is paramount to better clinical outcomes [6,7,8]. A key factor for a good prognosis and high survival rate is—besides the elimination of lifestyle risk factors—the early diagnosis of breast cancer. The improvement of current diagnostic tools, the development of new methods, and regular screenings have reduced cancer mortality dramatically, in combination with the overall survival rate and quality of life for patients [9,10].

In the pursuit of alternative and “outside-the-box” approaches for this and other detrimental diseases, medical research has begun to develop an interest in microgravity (μg) research [11,12,13]. The strain on the human body caused by the lack of gravitational force became apparent very early on in the age of spaceflight, as the technical challenges were overcome and astronauts started exploring the orbit and beyond and continued to do so [14,15,16,17]. Prolonged exposure of the human body to μg leads to multiple impairments in various physiological systems, among them the musculoskeletal system and bone metabolism, cardiovascular physiology, metabolic processes, and the microbiome [17,18,19,20,21,22,23,24,25]. These effects are observable not only on an organic but also on a cellular level, displaying changes in proteomic, genomic, and metabolomic profiles [26,27]. Studies of the angiovascular system in μg lead, among others, to advances in the understanding of wound healing and the three-dimensional growth of tumors [28,29,30,31].

Any kind of tissue growth is dependent on cell-cell communication, healthy or otherwise. Until roughly a decade ago, the only known methods of cell communication in multi-cellular systems were via direct cell contact or receptor-based signaling [32]. Since then, extracellular vesicles (EVs) have been discovered as an alternative way of cellular information exchange and have quickly gained attention. EVs comprise a family of membrane vesicles secreted from the majority, if not all, cells into the extracellular environment and functionally mediate cell-cell communication [33,34]. Three major types of extracellular vesicles can be distinguished: microvesicles, apoptotic bodies, and the so-called small EVs or exosomes. Exosomes are found in essentially all circulating body fluids, including blood, saliva, and urine, and can be distinguished from other EVs by their endosomal biogenesis, size, as well as several surfaces and internal markers [33,34,35,36,37,38,39]. The vesicles reflect their cell of origin in regard to their cargo and surface markers and, therefore, present as potential clinical biomarkers for a vast number of diseases [34,40,41]. With their properties to deliver their cargo and influence virtually any cell type, exosomes also promise a wide range of therapeutical applications [42]. Overcoming the technical challenges, starting with isolation and enrichment as well as proper characterization of these vesicles, the recent developments of very specific methods for small EV analysis enable the research community to address many questions about these fascinating cellular tools [43,44].

**Figure 1:**
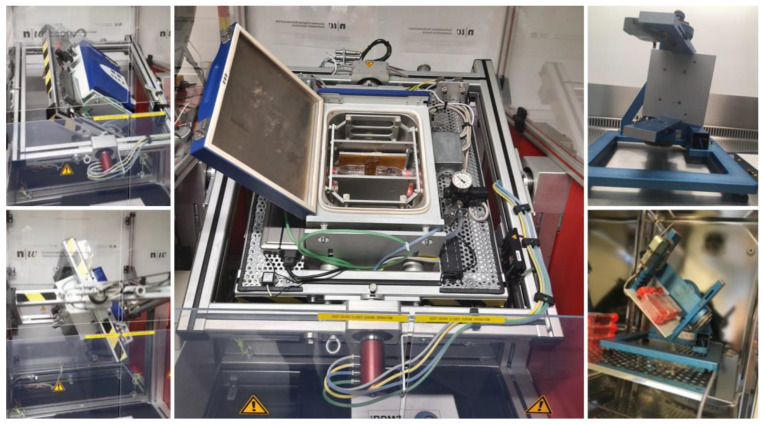
The spectrum of RPMs in our laboratory ranges from a small desktop RPM that remains in an incubator during operation (**right**) to a large RPM with an integrated incubator (**left** and **center**). The iRPM was designed and constructed by Jörg Seckler and Simon L. Wuest, Institute for Automation Engineering, University of Applied Sciences and Arts Northwestern Switzerland (FHNW), Brugg-Windisch, Aargau, Switzerland. The desktop RPM was purchased from Airbus Defense and Space (ADS), Leiden, The Netherlands.

Space travel has developed considerably in recent years in technology and the volume of spaceflights. The International Space Station (ISS) harbors research resources and supports multiple projects [45]. Nonetheless, with the apparent limitations in available space on the ISS, the still small amount of shuttle flights, and last but not least, the considerable costs, the opportunities for experiments in real microgravity (r-μg) are scarce. Therefore, a majority of experiments are being conducted on ground-based facilities under simulated microgravity (s-μg). To replicate μg satisfactorily, various devices have been developed, such as the random positioning machine (RPM, Figure 1) and clinostats [46,47,48,49,50]. These instruments are valuable resources for studying the influence of μg on a variety of cells under changing conditions and multiple time points. The experimental setup can be easily adapted and fairly rapidly, and the changes can be documented regularly during the time course of the experiment, which is a major advantage to experiments in r-μg [49]. The observed changes in cells following incubation within these devices are generally comparable to the cellular adaptations in r-μg, and even though they are not identical, they can often predict results gained during spaceflights [51].

Over the course of our investigations with several breast cancer cells exposed to both s-μg and r-μg, we have found that the tumor cells, incubated in s-μg, grow either adherently as a monolayer or as a 3D spheroid [52,53,54,55]. The spheroids model is a better in vitro representation of tumor growth in vivo; therefore, the cellular changes during the spheroid formation promise a deeper insight into tumor development and progression than ground-based cell culture experiments [54]. With cell-crosstalk in mind and the knowledge of the impact of small EVs on the cell-cell communication not only between tumor cells but also the tumor microenvironment and the preparation of the metastatic niche [34,56,57,58,59], the role of small EVs in the cellular response to μg is a factor that should not be underestimated. This research topic may provide a different angle and new findings worth exploring, also in the processes triggering 3D spheroid development.

Here we are investigating the changes in exosomal release and population in the breast cancer cell line MCF-7 following exposure to s-μg. We are aiming to learn whether the adaptive changes we observed in the CellBox-1 study will be mirrored. Ultimately, we would like to expand our knowledge on the three-dimensional growth of these and other tumor cells and how the communication between cells can be influenced to reduce tumorigenicity.

## 2. Results

The number of studies on EVs, and small EVs in particular, has risen quite rapidly following the first description of their importance in cell crosstalk [60,61]. The earliest experimental methods for EV isolation, namely differential centrifugation, filtration, and precipitation protocols, meanwhile proved to produce highly variable results in regard to yield, purity, and functionality [62,63,64]. To ensure reproducible experimental outcomes as much as to accommodate reliable research on the wide variety of source materials and investigational requirements, multiple methods have been developed to improve the isolation and characterization of EVs. In line with the volume of different source materials and cell types small and other EVs can be harvested from, the definitions and descriptions of exosomes were manifold, overlapping, and confusing. The International Society of Extracellular Vesicles (ISEV), therefore, divulged guidelines for the minimal information of studies in extracellular vesicles (MISEV), which includes a list of transmembrane proteins and cytosolic proteins with membrane-binding capacity anticipated to be present or absent in the various EV groups and recommended investigators to determine the expression of three or more of these proteins at least semi-quantitatively. Transmembrane proteins expected to be present in exosomal preparations include, among others, tetraspanins (CD9, CD63, CD81), integrins, and growth factor receptors. The group of expected or enriched cytosolic proteins includes endosome or membrane-binding proteins (TSG101) and signal transduction or scaffolding proteins (syntenin). Intracellular proteins that are absent or under-represented in exosomes but present in other types of EVs are proteins found in the endoplasmic reticulum (ER) (calnexin) or the Golgi apparatus (GM130).

As in our previous study on cell supernatants from the CellBox-1 experiment, the method of choice to investigate the supernatants of MCF-7 cells subjected to s-μg was SP-IRIS, specifically the ExoView™ system (Unchained Labs, Pleasanton, CA, USA). This way, human tetraspanin-positive exosomes are selectively captured on an antibody-coated chip directly from biofluids without a prior need to isolate and concentrate EVs. Additionally, the method is suitable for very small sample sizes, as high as 20 µL for unprocessed, not concentrated samples. As described previously, we used EV-TETRA-C ExoView Tetraspanin chip plates, which capture exosomes on spots that are separately pre-loaded with antibodies (Abs) to one of the tetraspanins CD9, CD63, and CD81, to assess the amount and population distribution of exosomes in the cell supernatants harvested following incubation of the cells on RPM for 5 and 10 d, as well as the corresponding 1 g controls. Via interferometric analysis, we were able to investigate the particle count and the size distribution of the given samples [43,65], and counter-staining the captured exosomes with fluorescent Abs to CD9, CD63, and CD81, we characterized the small EVs according to their transmembrane protein expression and identified the different populations present in the sample. Here, we describe the results we gained from these analyses.

### 2.1. Interferometric Analysis

#### 2.1.1. Particle Concentration

**Figure 2:**
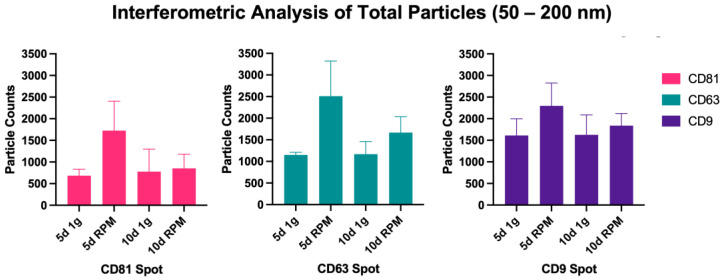
Particle count via Interferometry of particles >50 nm. Values were measured in triplicates of all three samples per experimental condition and timeline. Displayed are counts per capture spot.

The absolute number of captured particles in the size range of 50–200 nm was measured via interferometric analyses by scanning the tetraspanin spots (CD9, CD63, and CD81) as well as the IgG control, all in triplicates from three samples each of the following experimental condition: 5 d 1 g, 5 d RPM, 10 d 1 g and 10 d RPM. Figure 2 shows an overview of the particles captured in the size range from 50–200 nm. An increase in particle number following exposure to s-μg is found in both time points, with a considerably more pronounced increase at the 5 d timepoint. Table 1 details the average of all measured triplicates, their means, and standard deviation (SD). The number of small EVs captured at 5 d 1 g and 10 d 1 g is fairly constant at all three Tetraspanin spots (CD81: 5 d 1 g = 683.7; 10 d 1 g = 777.7; CD63: 5 d 1 g = 1151.3; 10 d 1 g = 169.3; CD9: 5 d 1 g = 1611.3; 10 d 1 g = 1625.0). The largest increase between the 1 g and RPM condition was seen at the 5 d time point: CD81: 5 d 1 g = 683.7; 5 d RPM = 1726.3; CD63: 5 d 1 g = 1151.3; 5 d RPM = 2511.0; CD9: 5 d 1 g = 1611.3; 5 d RPM = 2296.0, with none of the changes being significant. After 10 d incubation, there was still a slight increase in exosome particles after RPM exposure, but less distinct than after 5 d (CD81: 10 d 1 g = 777.7; 10 d RPM = 852.7; CD63: 10 d 1 g = 1169.3; 10 d RPM = 1667.7; CD9: 10 d 1 g = 1625.0; 10 d RPM = 1840.0). It is noteworthy that the amount of captured small EVs after 10 d on the RPM decreased considerably compared to 5 d exposure (Figure 2, Table 1).

#### 2.1.2. Particle Size Distribution

**Figure 3:**
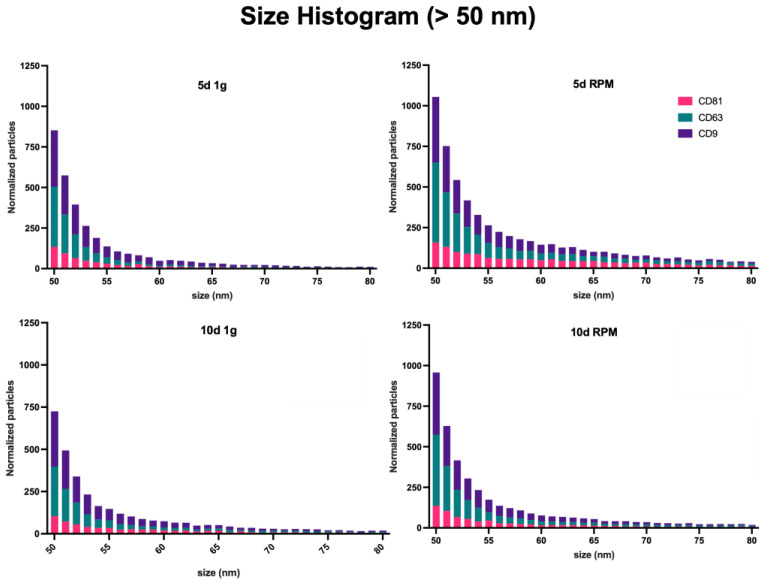
Particle size distribution by the interferometric analysis of all sample sets. Measurements were taken in triplicates from three samples each; the results were normalized with the IgG control, and the size range spans from 50–200 nm.

Measurement of the size distribution of the exosomes captured was equally done by interferometry. As this technique is limited to a range of 50–200 nm, it should be noted that a large amount of smaller particles, identified by the presence of a fluorescent signal and the absence of interferometric-based size measurement, are not included in these data. The number of these <50 nm particles will be included in the fluorimetric analysis. The majority of particles can be found within the range of 50–120 nm in all conditions and time points, as could be expected, and is consistent with the defined size range of exosomes (Figure 3, Supplemental Table S1) [62]. As described above, the difference between the particle number increase in this size range following RPM exposure vs. the 1 g control is more pointed after 5 d than 10 d (5 d 1 g = 1136.6; 5 d RPM = 2127.9; 10 d 1 g = 1173.1; 10 d RPM = 1433.2).

The mode of particles is at around 55 nm with an average of 519.9 particles at 5 d 1 g, 768.7 at 5 d RPM, 459.2 at 10 d 1 g, and 585.1 at 10 d RPM.

### 2.2. Fluorescent Analysis

#### 2.2.1. Total Fluorescent Particle Counts

As noted above, the interferometric analysis of the ExoView method is limited to the detection of particles sized 50 nm or larger, thus excluding a substantial part of exosomes. The results are still valuable to determine size distribution, but counterstaining the small EVs bound on the chip plate with fluorescent antibodies to the tetraspanins completes this initial analysis. Therefore, the combination of interferometric sizing and fluorometric detection allows the analysis of the entire EV content by detecting the size and evaluating the distribution of subpopulations defined by the presence of one or multiple exosomal markers.

**Figure 4:**
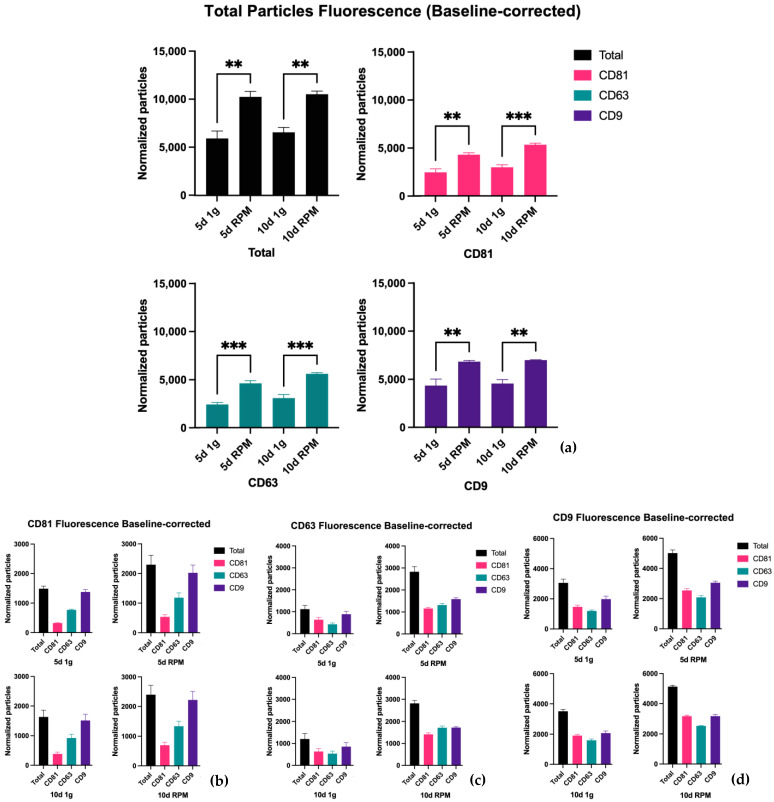
Particle number by fluorescence analysis via counterstain of captured small EVs with the tetraspanins CD81, CD63, and CD9. Measurements were taken in triplicates from three samples each; the results were normalized with the isotype control; the size includes particles below 50 nm. (**a**) Total number of particles from all capture spots. (**b**) Number of particles captured on the CD81 spot. (**c**) Number of particles captured on the CD63 spot. (**d**) Number of particles captured on the CD9 spot. ** is defined as *p* ≤ 0.01, *** as *p* ≤ 0.001.

The results of our examination of the total fluorescent particle counts with all three ABs on the three capture spots can be seen in Figure 4. Panel *a* displays the combined values of all three fluorescent tetraspanin ABs, and panels *b–d* displays the number of fluorescently stained particles on each capture spot. Comparing this to the analysis of the particles captured by interferometry, it can be stated that there is an increase in particle number after exposure of the BC cells to the RPM. The increase after 10 d RPM is on all three tetraspanin spots similar to the increase after 5 d RPM, suggesting that small EVs < 50 nm not only make up a large part of the total particle number but their expression is also much more influenced by exposure to s-μg. Statistically, the exposure of the MCF-7 cells to s-μg via RPM vs. the 1 g control shows significance in all comparisons and on all tetraspanin spots. The means of all samples (*n* = 3), the standard error of the means as well as the corresponding *p*-value are listed in Table 2 There is also a small but not significant increase in particle count after 10 d exposure vs. 5 d, in both the 1 g condition as well as after incubation of the cells on the RPM.

Looking at separate capture spots on the chip following the counterstain with the AB for CD81, CD63, and CD9, it becomes apparent that CD9 is the most dominantly expressed of the three tetraspanins (Figure 4a).

On the CD81 spot particularly, we observed that the EV count increased after s-μg at both time points; similarly, the surface protein expression of all three tetraspanins increased (Figure 4b, Table 3).

This pattern holds true for the CD63 spot as well, CD9 was the most expressed of the three surface proteins, but in contrast to the CD81 spot, the number of particles expressing the remaining two proteins showed a comparable value (Table 4). As prior, the particle count was elevated after incubation on the RPM compared to 1 g in both time points (Figure 4c).

On the CD9 spot again, EVs with CD9 expression displayed the highest particle count in all conditions and time points (Figure 4d, Table 5). This spot, though, has a considerably larger number expressing CD81 on its surface. All in all, the total exosome number captured on the CD9 spot is far greater than on either the CD81 or the CD63 spot (CD81 = 7823.1; CD63 = 7000.5; CD9 = 13,710.6).

#### 2.2.2. Colocalization Analysis

##### Single Tetraspanin Surface Expression—CD9, CD63, and CD81

**Figure 5:**
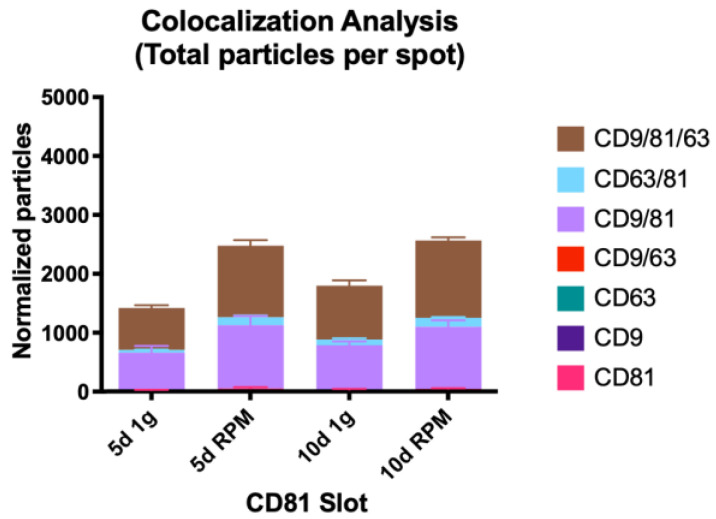
Visual representation of the colocalization analysis of the CD81 capture spot. All possible combinations are displayed: CD81, CD9/CD81, CD63/CD81 and CD9/CD63/CD81. None of the presented changes are significant.

The number of small EVs with an expression of one tetraspanin only, the previously described trend of an increased particle number post-exposure to s-μg continues apart from CD9 at the 10 d timepoint. Here the count of bound exosomes on the CD9 spot is slightly lower than after 10 d at 1 g with a mean of 912 (RPM) vs. 936.7 (1 g) (Figure 5, Table 6).

The count of CD81 only expressing exosomes is by far the lowest, the increase after 5 d RPM is more than double to 5 d 1 g (2.3-fold), whereas the increase following 10 d RPM is much more modest and even lower than after day 5 (1.4-fold).

**Figure 6:**
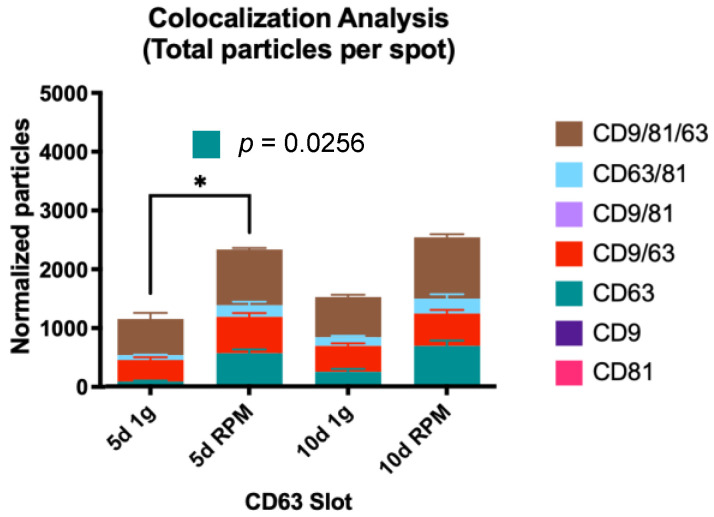
Visual representation of the colocalization analysis of the CD63 capture spot. All possible combinations are displayed: CD63, CD9/CD63, CD63/CD81 and CD9/CD63/CD81. The increase of CD63-only vesicles is significantly increased after 5 d of s-μg vs. 1 g. * is defined as *p* ≤ 0.05.

CD63-only expression vesicles show a stark increase between 5 d 1 g and 10 d 1 g; exposure of the MCF-7 cells to s-μg for 5 d increases the small EV number almost 7-fold, which is the only significant change in all populations (*p* = 0.0256). After the 10 d time point, the increase in the RPM sample is 2.8-fold (Figure 6, Table 7).

**Figure 7:**
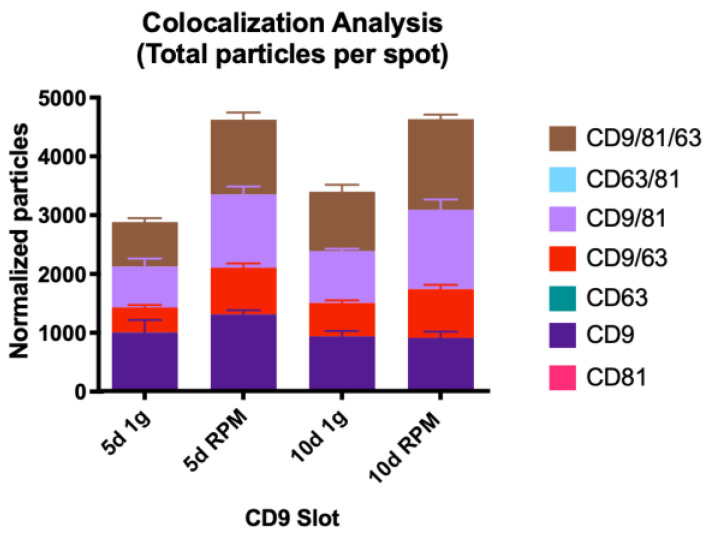
Visual representation of the colocalization analysis of the CD9 capture spot. All possible combinations are displayed: CD9, CD9/CD63, CD9/CD81 and CD9/CD63/CD81. None of the presented changes are significant.

EVs captured on the CD9 slot with single tetraspanin expression comprise the largest number of all. Overall, their number decreased after 10 d in both experimental conditions compared to 5 d; after 5 d, we recorded a 1.3-fold increase in particle number but a decrease of 0.03 after 10 d (Figure 7, Table 8).

##### Co-Expression of Two Tetraspanins—CD9/CD63, CD9/CD81, and CD63/CD81

When it comes to co-expression of CD9/CD63, the number of exosomes found on the CD63 spot compared to CD9 is slightly higher, and the EV numbers increase on both card spots after RPM incubation, spanning over a range of 1.3- to 1.9-fold (Table 7 and Table 8).

Tetraspanin co-expression of CD9/CD81 on the CD81 spot shows lower counts than on the CD9 spot, the fold-increase following the incubation under s-μg condition varies between 1.4 and 1.8 (Table 6 and Table 8).

Lastly, the number of EVs expressing CD63 and CD81 is low compared to the other tetraspanin combinations. Numbers on the CD63 spot are higher than on the CD81 card spot, with mean counts ranging from 46.3 to 254.3 and the fold increase varying from 1.55 to 2.95 (Table 6 and Table 7).

##### Co-Expression of All Three Tetraspanins—CD9/CD63/CD81

**Figure 8:**
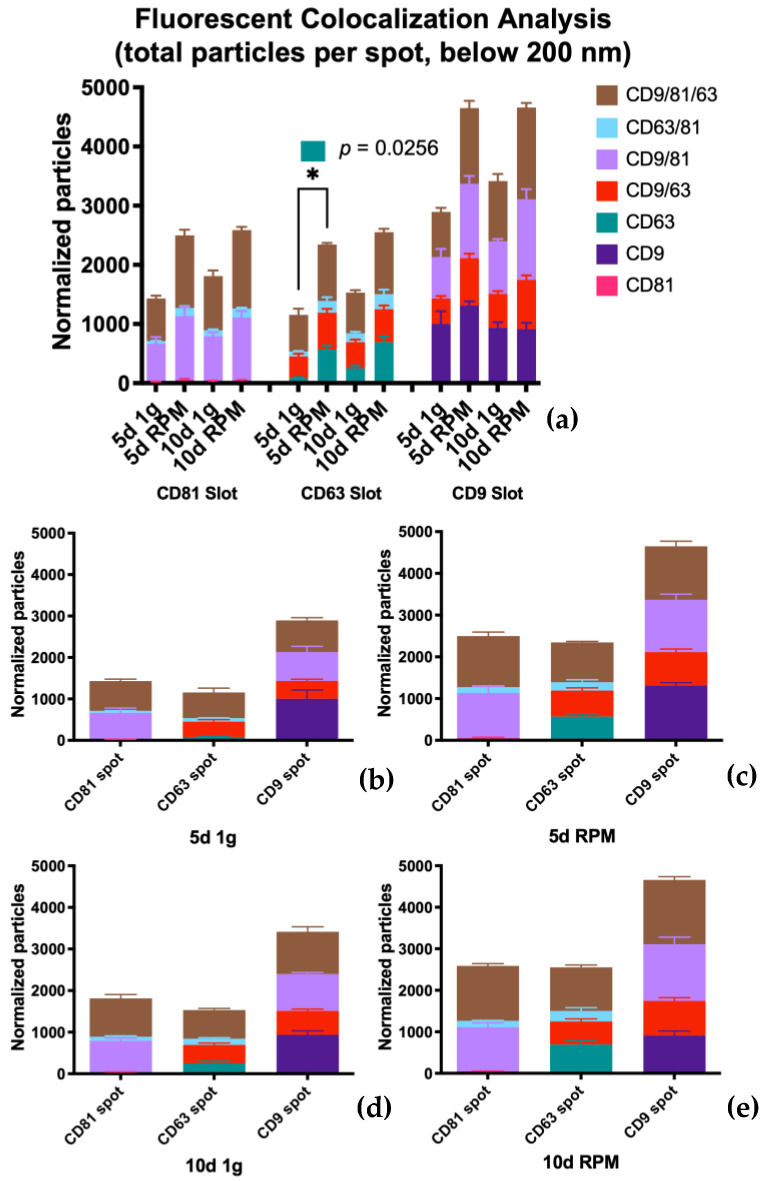
Fluorescence colocalization analysis via counterstain of captured small EVs with the tetraspanins CD81, CD63, and CD9. (**a**) Complete overview of exosome populations on the different tetraspanin spots at all experimental conditions. (**b**) Population detail of the three capture spots at 5 d 1 g. (**c**) Population detail of the three capture spots at 5 d RPM. (**d**) Population detail of the three capture spots at 10 d 1 g. (**e**) Population detail of the three capture spots at 10 d RPM.

The population expressing all three tetraspanins is the largest in all conditions over both time points. Comparing the three card spots, this set of exosomes captured on the CD63 spots provide the smallest group (average counts from 620.7–1049.7) with almost the same fold increases in the RPM group: 1.54 at 5 d and 1.52 at 10 d (Table 8, Figure 8). The small EVs with triple protein expression on the CD81 spot show a higher count; the average numbers range from 720.3 to 1325.3 with a fold change of 1.7 at 5 d and 1.44 at 10 d (Table 7, Figure 8). The largest group of exosomes is found on the CD9 spot, with values between 763.7 and 1554.0 on average and a fold change of 1.68 on 5 d and 1.53 on 10 d of s-μg (Table 7, Figure 8).

## 3. Discussion

Female breast cancer is a vast burden for patients and caregivers alike. The incidence rates are higher than ever, despite the considerable advances made in treatments and early discovery. The reasons why breast cancer cases are still on the rise are multifarious, be it the heterogeneity of the disease, which calls for special targeted or even personalized treatment options, the rise in lifestyle and environmental risk factors, genetic predisposition, or in some instances the lack of access to care [1,3,5,10,66]. The fast-paced development of new detection methods as well as new approaches to treatment though, have better clinical outcomes for breast cancer patients and improved their quality of life [66]. Despite these improvements, there is still a need to look further to discover new ways to better understand the development of this cancer and to find new opportunities for a cure.

Cancer cells and tumors, even more than every other living tissue, depend on cell-cell communication [56,67,68]. Besides the exchange of information via direct cell contact or receptor-based transmission, extracellular vesicles are the alternate option for cell communication [56,57,69,70]. In recent years, these EVs have emerged as the major player in information transfer during tumor growth, immune escape, TME education, progression, metastasis, and the preparation of the metastatic niche [56,57,70,71,72]. With their presence in virtually all biofluids, EVs are easily accessible biomarkers for cancers and other physiological maladies [42,69,73]. As biomolecules, they are very stable and thus a great resource to research changes in the number of secreted vesicles as well as in the contained cargo following varying physiological or experimental conditions [70,74,75,76,77]. Lastly, by altering the cargo and/or the expression of surface proteins of the EVs, they present themselves as ideal vesicles for personalized, targeted treatments for the majority of malignancies and other clinical pictures.

Our group has worked with various tumor cell lines, including breast cancer, and explored their genomic and proteomic changes after subjection to s- or r-μg. s-μg is a particularly useful tool in tumor research as several malignant cell lines will form 3-dimensional spheroids when incubated under μg conditions, which is a much better representation of a tumorigenic growth than in regular 2D cell culture [31,78,79]. To find supporting evidence of the results gained in previous experiments, we have also examined the changes in EV release in thyroid cancer cells following exposure to r-μg on the ISS [80].

In this current study, we examined the changes in exosome release in MCF-7 breast cancer cells following the subjection to s-μg for either 5 d or 10 d compared to the respective 1 g controls. As we did previously, we analyzed the small EV number, their size distribution, and the expression of the surface tetraspanins CD81, CD63, and CD9. Similar to the results we described in the study using r-μg, here we find an increased secretion of EVs following incubation to the RPM. The experimental time points were 5 d and 10 d; our results show a merely minimal increase in exosome numbers, if at all. This leads us to believe that the adaptive changes in the cells leading to a change in exosomal release occur fairly quickly upon the onset of s-μg. As mentioned previously, the interferometric analysis of small EVs is due to the limitation to vesicles larger than 50 nm lacking a large part of the entire exosome population. Still, through the size distribution we have shown that the particles captured on the card spots fall into the defined size range of exosomes with a mode of around 55 nm. Looking at the total particle counts after the fluorescent analysis of the bound vesicles, we can see that a very large part of small EVs is found in the group smaller than 50 nm. Additionally, the variance between the different samples is much lower, most likely due to the higher vesicle number. The fluorescence analysis shows significant changes in the release of exosomes following the cultivation on the RPM at both time points on every capture spot, whereas we have no significant changes to report when we analyze the populations in the 50–200 nm size range. The changes in the various populations expressing one, two, or all three tetraspanins seem to follow the release pattern of exosomes with increases in most populations following the exposure to s-μg. It seems noteworthy to point out that compared to our previous study, our current results are somewhat comparable regardless of the exposure to s-μg vs. r-μg and the use of breast cancer vs. thyroid cancer cells.

Of the three analyzed tetraspanins, small EVs with surface expression of CD9 are, compared to CD81 and CD63 populations, the largest group, both within single or coexpression. CD9 has been described as determining invasiveness and tumorigenicity in breast cancer cells [81]. Similarly, CD9 overexpression is implied to play a role in breast cancer chemoresistance and is considered a biomarker for various cancers [41,82,83,84]. CD63 expression is the second highest overall; this tetraspanin has been described as carrying both tumor suppressor as well as protumorigenic properties and is a predictor of poor prognosis in several cancers [85]. Just like CD9, CD63 plays a part in the regulation of breast cancer cell malignancy and the therapy resistance to tamoxifen [86,87]. Lastly, CD81 is the tetraspanin expressed in the lowest number of exosomes in the entire pool of populations. It does seem to hold the same properties, though, in breast cancer in regard to cell migration and proliferation as CD9 and CD63, and suppression of this surface protein is reported to reduce cancer invasion and metastasis [88,89].

**Figure 9:**
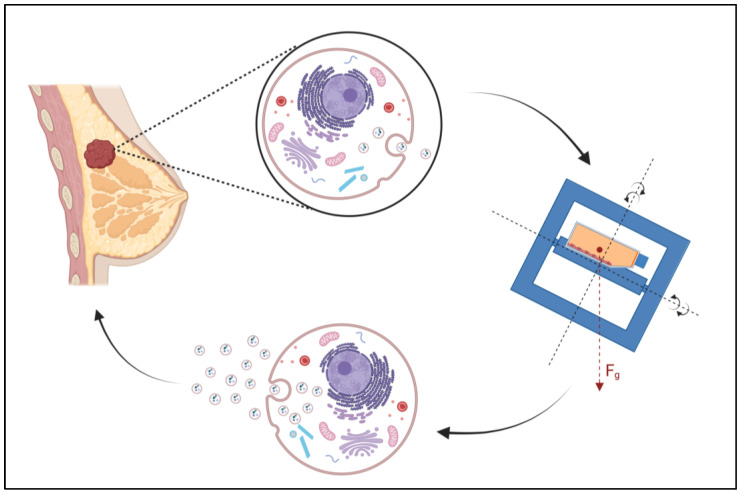
Graphical Abstract displaying the increase of small EV release in MCF-7 breast cancer cells following exposure to s-μg on a Random Positioning Machine (RPM) (Created with BioRender.com, license number: JM24RW7M01).

All in all, these results support our previous findings of the samples gained from the CellBox-1 experiment. The demonstrated increases in small EV release (Figure 9) suggest that the adaptive changes following μg conditions, either simulated or real, that we have described in previous studies on both the genomic or proteomic level, are somewhat mirrored in the cellular EV release and setup even though we cannot make any conclusions on possible adaptations to the cargo composition in the released vesicles. Whether the information transferred between cancer cells and the surrounding tumor microenvironment is restricted to the modification in the number of released vesicles or whether there is an actual variation in cargo needs to be determined. This study does demonstrate that the adaptive alterations within the cancer cells following changes in μg conditions are not only internal cellular events but that these adaptations are transmitted by vesicle-based cell-cell communication, which, therefore, may be a determining factor for the speed of the adaptive variations within the entire organism. This elevates the need for further investigations on the proteomic changes to the small EV cargo following exposure to μg. A recent proteomic analysis of both EVs and cells revealed a significant correlation with GTPases and proliferation of MDA-MB-231 cells in μg, and the authors conclude that EVs may be superior to cells in analyzing differentially expressed proteins, especially those that are down-regulated ones and usually unidentified or neglected in the analysis of intact cellular contents [90]. These are interesting new facets to this research area, which may aid in answering the question of how variations in the cargo setup add to the overall cellular response. We will attempt to address a variety of these questions in future follow-up studies.

## 4. Materials and Method

### 4.1. Cell Cultures

MCF-7 human breast adenocarcinoma cells were purchased from the American Type Culture Collection (ATCC) (Manassas, VA, USA). Cells were cultivated in RPMI 1640 medium (Life Technologies, Naerum, Denmark), supplemented with 10% fetal calf serum (FCS) (Biochrom, Berlin, Germany) and 1% penicillin/streptomycin (Biochrom) at 37 °C and 5% CO_2_. 1 × 10^6^ cells were seeded into T25 vented cell culture flasks (Sarstedt, Nümbrecht, Germany) and incubated ON to ensure proper attachment of the cells. Prior to the installation onto the RPM start and the experimental run, the flasks were filled completely with medium, air bubble-free. After 5 d, half of the flasks were removed, and cells and supernatant were harvested. The remaining flasks underwent a media change as described earlier [91], were incubated for another 5 d, and subsequently harvested. For the corresponding 1 g-controls, the flasks were placed and incubated adjacent to the RPM in the same incubator.

### 4.2. Random Positioning Machine

The desktop RPM (Airbus Defense and Space (ADS), Leiden, The Netherlands) was installed inside a standard incubator at 37 °C, 5% CO_2_. The RPM was operated in real random mode with random direction and interval and at a maximum speed of 12.5 revolutions per minute. Sample flasks to be tested were placed onto the middle frame with a maximal distance of 7 cm to the center of rotation, allowing a μg quality between 10^−4^ and 10^−2^ g, which is reached over time [49,67]. Corresponding static 1 g-controls, which were completely filled with medium, were placed next to the RPM in the same incubator (*n* = 15 samples for each group/run).

### 4.3. Exosome Harvest and Isolation

After the harvest, the cell supernatants of the RPM and control samples were subjected to an adjusted differential centrifugation protocol [92] using a swinging bucket rotor, consisting of two consecutive centrifugations, 300× *g* (10 min, 4 °C) followed by 2500× *g* (15 min, 4 °C, twice) to pellet cells, cell debris, and large vesicles. High-speed centrifugation was not necessary as the analysis via ExoView^®^ does not require upstream particle isolation. The collected supernatants were divided into 2 mL aliquots and stored at −80 °C until further analysis.

### 4.4. ExoView^®^ Kit Assay Procedure

For the capture and analysis of the CellBox-1 supernatants, the EV-TETRA-C ExoView Tetraspanin Kit (Unchained Labs, Pleasanton, CA, USA) was used, with all samples processed and stained according to the manufacturer’s protocol. The spots on the chip plates were coated with antibodies for the tetraspanins CD9, CD81, and CD63, as well as a negative IgG control, all in triplicates. In short, the samples, 0.5 uL, were diluted to 10 uL in PBS and incubated overnight, in a 1:2 dilution with the provided buffer (Solution A), on the sealed chip plate in order to capture the exosomes present. The incubation was followed by three wash steps prior to the surface membrane immunofluorescence staining. An antibody mixture containing fluorescently labeled anti-CD9 (CF^®^ 488A), anti-CD81 (CF^®^ 555), and anti-CD63 (CF^®^ 647) was pipetted onto the chip and incubated for 1 h on an orbital shaker, blocked from light exposure. Three wash steps with the supplied wash buffer, and two washes with DI water for 3 min each (RT, shaking, light omitted) followed this incubation. Subsequently, the chip plates were dried and scanned as described below.

### 4.5. Digital Detection of Exosomes

The chip plates were scanned using the ExoView R100 (Unchained labs, Pleasanton, CA, USA) in combination with the ExoViewer software. All the chip plates were pre-scanned prior to the exosome capture to obtain a baseline signal; then, the exosome-laden chips were scanned identically positioned on the stage platform. The analysis was visualized with the ExoViewer software, resulting in the EV count, size distribution, and colocalization of EV subpopulations.

### 4.6. Statistical Analysis

The total counts of numbers were analyzed via the unpaired t-test, comparing all the samples from either the GM or FM group respective to the capture spot on the chip plate, CD9, CD63, or CD81. The analysis was conducted using the GraphPad Prism 9 software (GraphPad Software, San Diego, CA, USA).

## 5. Conclusions

Despite advances in diagnosis and treatment, breast cancer is one of the most debilitating and deadly diseases of our time. Current knowledge about the importance of cell communication during all stages of tumor development, growth, and metastasis point to the role that extracellular vesicles can play in learning about and fighting all cancers. Applying s-μg to breast cancer cells leads to an improved in vitro model to study the changes in small EVs, among others. Investigations like this will extend our knowledge of cell communication in the tumor microenvironment and may result in breakthrough therapies to cure breast cancer.

## Figures and Tables

**Table 1:** Particle counts in averages per sample.

	**CD81**				
	**A**	**B**	**C**	**Mean**	**SD**
5 d 1 g	973.0	484.0	594.0	683.7	256.5
5 d RPM	2580.0	2211.0	388.0	1726.3	1173.6
10 d 1 g	313.0	1813.0	207.0	777.7	898.2
10 d RPM	552.0	1506.0	500.0	852.7	566.4
	**CD63**				
	**A**	**B**	**C**	**Mean**	**SD**
5 d 1 g	1223.0	1029.0	1202.0	1151.3	106.5
5 d RPM	3081.0	3537.0	915.0	2511.0	1400.9
10 d 1 g	1020.0	1728.0	760.0	1169.3	501.0
10 d RPM	1236.0	2394.0	1373.0	1667.7	632.7
	**CD9**				
	**A**	**B**	**C**	**Mean**	**SD**
5 d 1 g	2293.0	955.0	1586.0	1611.3	669.4
5 d RPM	2892.0	2756.0	1240.0	2296.0	917.0
10 d 1 g	1061.0	2539.0	1275.0	1625.0	798.7
10 d RPM	1530.0	2394.0	1596.0	1840.0	480.9

**Table 2:** Mean particle counts, standard errors of the mean, and *p* values.

Total			
	**Mean**	**SE**	***p* Value**
5 d 1 g	5929	1341	0.0013
5 d RPM	10,260	987.8	
10 d 1 g	6570	882.7	0.003
10 d RPM	10,529	572.1	
CD81			
	**Mean**	**SE**	***p* Value**
5 d 1 g	2462	362.8	0.0020
5 d RPM	4297	190.7	
10 d 1 g	2976	262.2	0.0004
10 d RPM	5327	163.7	
CD63			
	**Mean**	**SE**	***p* Value**
5 d 1 g	2416	217.7	0.0006
5 d RPM	4624	267.5	
10 d 1 g	3076	364.4	0.0002
10 d RPM	5606	129.4	
CD9			
	**Mean**	**SE**	***p* Value**
5 d 1 g	4350	671.9	0.0047
5 d RPM	6828	126.6	
10 d 1 g	4554	412.7	0.0052
10 d RPM	6988	40.8	

**Table 3:** Average fluorescent particle analysis of the CD81 card spot.

CD81				
	Total	CD81	CD63	CD9
5 d 1 g	1489.9	323.2	770.0	1379.2
5 d RPM	2299.1	535.7	1184.0	2022.7
10 d 1 g	1636.4	380.1	920.0	1516.9
10 d RPM	2397.7	697.1	1338.7	2221.7

**Table 4:** Average fluorescent particle analysis of the CD63 card spot.

CD63				
	Total	CD81	CD63	CD9
5 d 1 g	1119.4	639.6	429.9	887.8
5 d RPM	2826.8	1157.2	1314.4	1583.4
10 d 1 g	1201.3	631.8	533.3	855.9
10 d RPM	1853.0	623.5	927.2	1495.4

**Table 5:** Average fluorescent particle analysis of the CD9 card spot.

CD9				
	Total	CD81	CD63	CD9
5 d 1 g	3059.0	1468.6	1196.2	1982.8
5 d RPM	2011.9	904.1	880.2	1361.1
10 d 1 g	3507.6	1906.4	1592.4	2071.4
10 d RPM	5132.1	3176.8	2533.6	3182.9

**Table 6:** Colocalization analysis of the CD81 card spot.

CD81								
	CD81	Fold Change	CD9/CD81	Fold Change	CD63/CD81	Fold Change	CD9/CD63/CD81	Fold Change
5 d 1 g	23.0	2.27	641.0	1.69	46.3	2.95	720.3	1.70
5 d RPM	52.3		1082.7		136.7		1225.7	
10 d 1 g	32.7	1.44	758.7	1.40	100.3	1.55	919.7	1.44
10 d RPM	47.0		1060.0		155.7		1325.3	

**Table 7:** Colocalization analysis of the CD63 card spot.

CD63								
	CD63	Fold Change	CD9/CD63	Fold Change	CD63/CD81	Fold Change	CD9/CD63/CD81	Fold Change
5 d 1 g	82.3	6.89	369.7	1.69	83.3	2.41	620.7	1.54
5 d RPM	567.3		623.3		200.7		953.7	
10 d 1 g	250.3	2.77	443.3	1.25	149.3	1.70	688.3	1.52
10 d RPM	693.3		555.7		254.3		1049.7	

**Table 8:** Colocalization analysis of the CD9 card spot.

CD9								
	CD9	Fold Change	CD9/CD63	Fold Change	CD9/CD81	Fold Change	CD9/CD63/CD81	Fold Change
5 d 1 g	1003.3	1.31	429.7	1.86	699.3	1.79	763.7	1.68
5 d RPM	1313.0		799.7		1254.7		1283.3	
10 d 1 g	936.7	0.97	573.7	1.45	887.3	1.53	1018.0	1.53
10 d RPM	912.0		832.7		1361.0		1554.0	

## Data Availability

Not applicable.

## Supplementary Materials

The following supporting information can be downloaded at: https://www.mdpi.com/article/10.3390/ijms232416095/s1.

## Abbreviations

AB	Antibody
EMT	Epithelial to Mesenchymal Transition
EV	Extracellular Vesicle
GM	Ground Module
FM	Flight Module
ILV	Intraluminal Vesicle
ISS	International Space Station
MVBs	Multivesicular Bodies
NTA	Nanoparticle Tracking Analysis
ON	Overnight
RPM	Random Positioning Machine
RT	Room temperature
r-μg	Real Microgravity
SD	Standard Deviation
SP-IRIS	Single Particle Interferometric Reflectance Imaging Sensor
s-μg	Simulated Microgravity
TME	Tumor Microenvironment

## References

[1] Sung H., Ferlay J., Siegel R.L., Laversanne M., Soerjomataram I., Jemal A., Bray F. (2021). Global Cancer Statistics 2020: GLOBOCAN Estimates of Incidence and Mortality Worldwide for 36 Cancers in 185 Countries. CA Cancer J. Clin..

[2] Wild C., Weiderpass E., Stewart B. (2020). World Cancer Report: Cancer Research for Cancer Prevention.

[3] Brinton L.A., Gierach G.L., Thun M.J., Linet M.S., Cerhan J.R., Haiman C.A., Schottenfeld D. (2018). Breast Cancer. Cancer Epidemiology and Prevention.

[4] Shiovitz S., Korde L.A. (2015). Genetics of breast cancer: A topic in evolution. Ann. Oncol..

[5] Picon-Ruiz M., Morata-Tarifa C., Valle-Goffin J.J., Friedman E.R., Slingerland J.M. (2017). Obesity and adverse breast cancer risk and outcome: Mechanistic insights and strategies for intervention. CA Cancer J. Clin..

[6] Waks A.G., Winer E.P. (2019). Breast Cancer Treatment: A Review. JAMA.

[7] Yeo S.K., Guan J.L. (2017). Breast Cancer: Multiple Subtypes within a Tumor?. Trends Cancer.

[8] Li J., Chen Z., Su K., Zeng J. (2015). Clinicopathological classification and traditional prognostic indicators of breast cancer. Int. J. Clin. Exp. Pathol..

[9] Chen L., Jiang N., Wu Y. (2020). Application and Analysis of Biomedical Imaging Technology in Early Diagnosis of Breast Cancer. Methods Mol. Biol..

[10] Merino Bonilla J.A., Torres Tabanera M., Ros Mendoza L.H. (2017). Breast cancer in the 21st century: From early detection to new therapies. Radiologia.

[11] Grassi B. (2018). Bed Rest Studies as Analogs of Conditions Encountered in Space and in Diseases. Med. Sci. Sport. Exerc..

[12] Droppert P.M. (1993). A review of muscle atrophy in microgravity and during prolonged bed rest. J. Br. Interplanet. Soc..

[13] Hargens A.R., Vico L. (2016). Long-duration bed rest as an analog to microgravity. J. Appl. Physiol..

[14] Garrett-Bakelman F.E., Darshi M., Green S.J., Gur R.C., Lin L., Macias B.R., McKenna M.J., Meydan C., Mishra T., Nasrini J. (2019). The NASA Twins Study: A multidimensional analysis of a year-long human spaceflight. Science.

[15] White R.J., Averner M. (2001). Humans in space. Nature.

[16] White R.J. (1998). Weightlessness and the human body. Sci. Am..

[17] BALDWIN K.M. (1996). Effect of spaceflight on the functional, biochemical, and metabolic properties of skeletal muscle. Med. Sci. Sport. Exerc..

[18] Strollo F., Bontinc S.L. (1999). Chapter 4 Hormonal Changes in Humans During Spaceflight. Advances in Space Biology and Medicine.

[19] Taylor G.R. (1993). Overview of spaceflight immunology studies. J. Leukoc. Biol..

[20] Battista N., Meloni M.A., Bari M., Mastrangelo N., Galleri G., Rapino C., Dainese E., Agrò A.F., Pippia P., Maccarrone M. (2012). 5-Lipoxygenase-dependent apoptosis of human lymphocytes in the International Space Station: Data from the ROALD experiment. FASEB J..

[21] Baldwin K.M., White T.P., Arnaud S.B., Edgerton V.R., Kraemer W.J., Kram R., Raab-Cullen D., Snow C.M. (1996). Musculoskeletal adaptations to weightlessness and development of effective countermeasures. Med. Sci. Sport. Exerc..

[22] Shen M., Frishman W.H. (2019). Effects of Spaceflight on Cardiovascular Physiology and Health. Cardiol. Rev..

[23] Grimm D., Grosse J., Wehland M., Mann V., Reseland J.E., Sundaresan A., Corydon T.J. (2016). The impact of microgravity on bone in humans. Bone.

[24] Thiel C.S., Vahlensieck C., Bradley T., Tauber S., Lehmann M., Ullrich O. (2021). Metabolic Dynamics in Short- and Long-Term Microgravity in Human Primary Macrophages. Int. J. Mol. Sci..

[25] Siddiqui R., Akbar N., Khan N.A. (2021). Gut microbiome and human health under the space environment. J. Appl. Microbiol..

[26] Nichols H.L., Zhang N., Wen X. (2006). Proteomics and genomics of microgravity. Physiol. Genom..

[27] Michaletti A., Gioia M., Tarantino U., Zolla L. (2017). Effects of microgravity on osteoblast mitochondria: A proteomic and metabolomics profile. Sci. Rep..

[28] Morbidelli L., Genah S., Cialdai F. (2021). Effect of Microgravity on Endothelial Cell Function, Angiogenesis, and Vessel Remodeling During Wound Healing. Front. Bioeng. Biotechnol..

[29] Siddiqui R., Qaisar R., Goswami N., Khan N.A., Elmoselhi A. (2021). Effect of Microgravity Environment on Gut Microbiome and Angiogenesis. Life.

[30] Gregg R.K. (2021). Implications of microgravity-induced cell signaling alterations upon cancer cell growth, invasiveness, metastatic potential, and control by host immunity. Int. Rev. Cell Mol. Biol..

[31] Kopp S., Warnke E., Wehland M., Aleshcheva G., Magnusson N.E., Hemmersbach R., Corydon T.J., Bauer J., Infanger M., Grimm D. (2015). Mechanisms of three-dimensional growth of thyroid cells during long-term simulated microgravity. Sci. Rep..

[32] Cooper G.M. (2000). The Cell: A Molecular Approach.

[33] Colombo M., Raposo G., Thery C. (2014). Biogenesis, secretion, and intercellular interactions of exosomes and other extracellular vesicles. Annu. Rev. Cell Dev. Biol..

[34] Abels E.R., Breakefield X.O. (2016). Introduction to Extracellular Vesicles: Biogenesis, RNA Cargo Selection, Content, Release, and Uptake. Cell Mol. Neurobiol..

[35] Orozco A.F., Lewis D.E. (2010). Flow cytometric analysis of circulating microparticles in plasma. Cytom. A.

[36] Gonzales P.A., Pisitkun T., Hoffert J.D., Tchapyjnikov D., Star R.A., Kleta R., Wang N.S., Knepper M.A. (2009). Large-scale proteomics and phosphoproteomics of urinary exosomes. J. Am. Soc. Nephrol..

[37] Sharma S., Gillespie B.M., Palanisamy V., Gimzewski J.K. (2011). Quantitative Nanostructural and Single-Molecule Force Spectroscopy Biomolecular Analysis of Human-Saliva-Derived Exosomes. Langmuir.

[38] Théry C., Witwer K.W., Aikawa E., Alcaraz M.J., Anderson J.D., Andriantsitohaina R., Antoniou A., Arab T., Archer F., Atkin-Smith G.K. (2018). Minimal information for studies of extracellular vesicles 2018 (MISEV2018): A position statement of the International Society for Extracellular Vesicles and update of the MISEV2014 guidelines. J. Extracell. Vesicles.

[39] Lötvall J., Hill A.F., Hochberg F., Buzás E.I., Di Vizio D., Gardiner C., Gho Y.S., Kurochkin I.V., Mathivanan S., Quesenberry P. (2014). Minimal experimental requirements for definition of extracellular vesicles and their functions: A position statement from the International Society for Extracellular Vesicles. J. Extracell. Vesicles.

[40] Raposo G., Stoorvogel W. (2013). Extracellular vesicles: Exosomes, microvesicles, and friends. J. Cell Biol..

[41] Hoshino A., Kim H.S., Bojmar L., Gyan K.E., Cioffi M., Hernandez J., Zambirinis C.P., Rodrigues G., Molina H., Heissel S. (2020). Extracellular Vesicle and Particle Biomarkers Define Multiple Human Cancers. Cell.

[42] Barile L., Vassalli G. (2017). Exosomes: Therapy delivery tools and biomarkers of diseases. Pharmacol. Ther..

[43] Daaboul G.G., Gagni P., Benussi L., Bettotti P., Ciani M., Cretich M., Freedman D.S., Ghidoni R., Ozkumur A.Y., Piotto C. (2016). Digital Detection of Exosomes by Interferometric Imaging. Sci. Rep..

[44] Chen J., Li P., Zhang T., Xu Z., Huang X., Wang R., Du L. (2022). Review on Strategies and Technologies for Exosome Isolation and Purification. Front. Bioeng. Biotechnol..

[45] Kramer C.D., Kalla E.M. The challenge of designing biomedical equipment during human research for long duration low-gravity NASA missions. Proceedings of the 1997 16 Southern Biomedical Engineering Conference.

[46] Hoson T., Kamisaka S., Masuda Y., Yamashita M., Buchen B. (1997). Evaluation of the three-dimensional clinostat as a simulator of weightlessness. Planta.

[47] Maccarrone M., Battista N., Meloni M., Bari M., Galleri G., Pippia P., Cogoli A., Finazzi-Agrò A. (2003). Creating conditions similar to those that occur during exposure of cells to microgravity induces apoptosis in human lymphocytes by 5-lipoxygenase-mediated mitochondrial uncoupling and cytochrome c release. J. Leukoc. Biol..

[48] Herranz R., Anken R., Boonstra J., Braun M., Christianen P.C.M., de Geest M., Hauslage J., Hilbig R., Hill R.J.A., Lebert M. (2013). Ground-based facilities for simulation of microgravity: Organism-specific recommendations for their use, and recommended terminology. Astrobiology.

[49] van Loon J.J.W.A. (2007). Some history and use of the random positioning machine, RPM, in gravity related research. Adv. Space Res..

[50] Pietsch J., Bauer J., Egli M., Infanger M., Wise P., Ulbrich C., Grimm D. (2011). The effects of weightlessness on the human organism and mammalian cells. Curr. Mol. Med..

[51] Schwarzenberg M., Pippia P., Meloni M.A., Cossu G., Cogoli-Greuter M., Cogoli A. (1999). Signal transduction in T lymphocytes—A comparison of the data from space, the free fall machine and the random positioning machine. Adv. Space Res..

[52] Nassef M.Z., Kopp S., Wehland M., Melnik D., Sahana J., Krüger M., Corydon T.J., Oltmann H., Schmitz B., Schütte A. (2019). Real Microgravity Influences the Cytoskeleton and Focal Adhesions in Human Breast Cancer Cells. Int. J. Mol. Sci..

[53] Nassef M.Z., Kopp S., Melnik D., Corydon T.J., Sahana J., Krüger M., Wehland M., Bauer T.J., Liemersdorf C., Hemmersbach R. (2019). Short-Term Microgravity Influences Cell Adhesion in Human Breast Cancer Cells. Int. J. Mol. Sci..

[54] Grimm D., Schulz H., Krüger M., Cortés-Sánchez J.L., Egli M., Kraus A., Sahana J., Corydon T.J., Hemmersbach R., Wise P.M. (2022). The Fight against Cancer by Microgravity: The Multicellular Spheroid as a Metastasis Model. Int J Mol Sci.

[55] Bauer J., Wehland M., Infanger M., Grimm D., Gombocz E. (2018). Semantic Analysis of Posttranslational Modification of Proteins Accumulated in Thyroid Cancer Cells Exposed to Simulated Microgravity. Int. J. Mol. Sci..

[56] Becker A., Thakur B.K., Weiss J.M., Kim H.S., Peinado H., Lyden D. (2016). Extracellular Vesicles in Cancer: Cell-to-Cell Mediators of Metastasis. Cancer Cell.

[57] Costa-Silva B., Aiello N.M., Ocean A.J., Singh S., Zhang H., Thakur Basant K., Becker A., Hoshino A., Mark M.T., Molina H. (2015). Pancreatic cancer exosomes initiate pre-metastatic niche formation in the liver. Nat. Cell Biol..

[58] Feng W., Dean D.C., Hornicek F.J., Shi H., Duan Z. (2019). Exosomes promote pre-metastatic niche formation in ovarian cancer. Mol. Cancer.

[59] Nogués L., Benito-Martin A., Hergueta-Redondo M., Peinado H. (2018). The influence of tumour-derived extracellular vesicles on local and distal metastatic dissemination. Mol. Asp. Med..

[60] Harding C., Stahl P. (1983). Transferrin recycling in reticulocytes: pH and iron are important determinants of ligand binding and processing. Biochem. Biophys. Res. Commun..

[61] Pan B.T., Johnstone R.M. (1983). Fate of the transferrin receptor during maturation of sheep reticulocytes in vitro: Selective externalization of the receptor. Cell.

[62] Au Lässer C., Au Eldh M., Au Lötvall J. (2012). Isolation and Characterization of RNA-Containing Exosomes. J. Vis. Exp. JoVE.

[63] Shu S., Yang Y., Allen C.L., Hurley E., Tung K.H., Minderman H., Wu Y., Ernstoff M.S. (2020). Purity and yield of melanoma exosomes are dependent on isolation method. J. Extracell. Vesicles.

[64] Monguió-Tortajada M., Gálvez-Montón C., Bayes-Genis A., Roura S., Borràs F.E. (2019). Extracellular vesicle isolation methods: Rising impact of size-exclusion chromatography. Cell Mol. Life Sci..

[65] Avci O., Ünlü N.L., Özkumur A.Y., Ünlü M.S. (2015). Interferometric Reflectance Imaging Sensor (IRIS)—A Platform Technology for Multiplexed Diagnostics and Digital Detection. Sensors.

[66] Sun Y.S., Zhao Z., Yang Z.N., Xu F., Lu H.J., Zhu Z.Y., Shi W., Jiang J., Yao P.P., Zhu H.P. (2017). Risk Factors and Preventions of Breast Cancer. Int. J. Biol. Sci..

[67] Warnke E., Pietsch J., Wehland M., Bauer J., Infanger M., Görög M., Hemmersbach R., Braun M., Ma X., Sahana J. (2014). Spheroid formation of human thyroid cancer cells under simulated microgravity: A possible role of CTGF and CAV1. Cell Commun. Signal..

[68] Wen S.W., Sceneay J., Lima L.G., Wong C.S., Becker M., Krumeich S., Lobb R.J., Castillo V., Wong K.N., Ellis S. (2016). The Biodistribution and Immune Suppressive Effects of Breast Cancer-Derived Exosomes. Cancer Res..

[69] Cui S., Cheng Z., Qin W., Jiang L. (2018). Exosomes as a liquid biopsy for lung cancer. Lung Cancer.

[70] D’Asti E., Chennakrishnaiah S., Lee T.H., Rak J. (2016). Extracellular Vesicles in Brain Tumor Progression. Cell Mol. Neurobiol..

[71] Alderton G.K. (2012). Metastasis. Exosomes drive premetastatic niche formation. Nat. Rev. Cancer.

[72] Alimirzaie S., Bagherzadeh M., Akbari M.R. (2019). Liquid biopsy in breast cancer: A comprehensive review. Clin. Genet..

[73] Bezdan D., Grigorev K., Meydan C., Pelissier Vatter F.A., Cioffi M., Rao V., MacKay M., Nakahira K., Burnham P., Afshinnekoo E. (2020). Cell-free DNA (cfDNA) and Exosome Profiling from a Year-Long Human Spaceflight Reveals Circulating Biomarkers. iScience.

[74] Herrmann M., Engelke K., Ebert R., Müller-Deubert S., Rudert M., Ziouti F., Jundt F., Felsenberg D., Jakob F. (2020). Interactions between Muscle and Bone-Where Physics Meets Biology. Biomolecules.

[75] Hannafon B.N., Trigoso Y.D., Calloway C.L., Zhao Y.D., Lum D.H., Welm A.L., Zhao Z.J., Blick K.E., Dooley W.C., Ding W.Q. (2016). Plasma exosome microRNAs are indicative of breast cancer. Breast Cancer Res..

[76] Joyce D.P., Kerin M.J., Dwyer R.M. (2016). Exosome-encapsulated microRNAs as circulating biomarkers for breast cancer. Int. J. Cancer.

[77] Zhao L., Gu C., Gan Y., Shao L., Chen H., Zhu H. (2020). Exosome-mediated siRNA delivery to suppress postoperative breast cancer metastasis. J. Control Release.

[78] Nassef M.Z., Melnik D., Kopp S., Sahana J., Infanger M., Lützenberg R., Relja B., Wehland M., Grimm D., Krüger M. (2020). Breast Cancer Cells in Microgravity: New Aspects for Cancer Research. Int. J. Mol. Sci..

[79] Riwaldt S., Bauer J., Wehland M., Slumstrup L., Kopp S., Warnke E., Dittrich A., Magnusson N.E., Pietsch J., Corydon T.J. (2016). Pathways Regulating Spheroid Formation of Human Follicular Thyroid Cancer Cells under Simulated Microgravity Conditions: A Genetic Approach. Int. J. Mol. Sci..

[80] Wise P.M., Neviani P., Riwaldt S., Corydon T.J., Wehland M., Braun M., Krüger M., Infanger M., Grimm D. (2021). Changes in Exosome Release in Thyroid Cancer Cells after Prolonged Exposure to Real Microgravity in Space. Int. J. Mol. Sci..

[81] Rappa G., Green T.M., Karbanová J., Corbeil D., Lorico A. (2015). Tetraspanin CD9 determines invasiveness and tumorigenicity of human breast cancer cells. Oncotarget.

[82] Kischel P., Bellahcene A., Deux B., Lamour V., Dobson R., De Pauw E., Clezardin P., Castronovo V. (2012). Overexpression of CD9 in human breast cancer cells promotes the development of bone metastases. Anticancer Res..

[83] Pacienza N., Yannarelli G. (2019). CD9: A possible clue into breast cancer chemoresistance. Oncotarget.

[84] Ullah M., Akbar A., Ng N.N., Concepcion W., Thakor A.S. (2019). Mesenchymal stem cells confer chemoresistance in breast cancer via a CD9 dependent mechanism. Oncotarget.

[85] Kaprio T., Hagström J., Andersson L.C., Haglund C. (2020). Tetraspanin CD63 independently predicts poor prognosis in colorectal cancer. Histol. Histopathol..

[86] Gao Y., Li X., Zeng C., Liu C., Hao Q., Li W., Zhang K., Zhang W., Wang S., Zhao H. (2020). CD63(+) Cancer-Associated Fibroblasts Confer Tamoxifen Resistance to Breast Cancer Cells through Exosomal miR-22. Adv. Sci..

[87] Tominaga N., Hagiwara K., Kosaka N., Honma K., Nakagama H., Ochiya T. (2014). RPN2-mediated glycosylation of tetraspanin CD63 regulates breast cancer cell malignancy. Mol. Cancer.

[88] Vences-Catalán F., Rajapaksa R., Kuo C.C., Miller C.L., Lee A., Ramani V.C., Jeffrey S.S., Levy R., Levy S. (2021). Targeting the tetraspanin CD81 reduces cancer invasion and metastasis. Proc. Natl. Acad. Sci. USA.

[89] Zhang N., Zuo L., Zheng H., Li G., Hu X. (2018). Increased Expression of CD81 in Breast Cancer Tissue is Associated with Reduced Patient Prognosis and Increased Cell Migration and Proliferation in MDA-MB-231 and MDA-MB-435S Human Breast Cancer Cell Lines In Vitro. Med. Sci. Monit..

[90] Chen Y., Xue F., Russo A., Wan Y. (2021). Proteomic Analysis of Extracellular Vesicles Derived from MDA-MB-231 Cells in Microgravity. Protein J..

[91] Corydon T.J., Mann V., Slumstrup L., Kopp S., Sahana J., Askou A.L., Magnusson N.E., Echegoyen D., Bek T., Sundaresan A. (2016). Reduced Expression of Cytoskeletal and Extracellular Matrix Genes in Human Adult Retinal Pigment Epithelium Cells Exposed to Simulated Microgravity. Cell. Physiol. Biochem..

[92] Théry C., Amigorena S., Raposo G., Clayton A. (2006). Isolation and characterization of exosomes from cell culture supernatants and biological fluids. Curr. Protoc. Cell Biol..

