# Supplementary material for: Prolonged Exposure to Simulated Microgravity Changes Release of Small Extracellular Vesicle in Breast Cancer Cells
Source: Int J Mol Sci. 2022 Dec 17;23(24):16095. doi: 10.3390/ijms232416095 (PMC9781806; doi:10.3390/ijms232416095)
Supplement: Supplementary file 1 [file ijms-23-16095-s001.zip › ijms-2102586-supplementary.pdf]

Supplemental Table S1. Particle size distribution baseline corrected, all sample sets (50 – 200nm).

5d 1*g*									
	**CD63**			**CD81**			**CD9**		
**Size (nm)**	**A**	**B**	**C**	**A**	**B**	**C**	**A**	**B**	**C**
**50**	372	354	385	200	87	114	461	241	343
**55**	634	473	589	444	181	210	1041	425	682
**60**	99	76	88	116	77	83	322	112	224
**65**	43	35	45	72	33	57	165	63	130
**70**	22	18	21	36	33	33	101	31	76
**75**	10	16	17	24	22	26	65	20	48
**80**	7	12	7	19	17	20	43	11	22
**85**	4	8	5	9	8	8	28	12	10
**90**	3	5	6	9	7	5	14	9	14
**95**	6	7	6	7	5	4	9	7	9
**100**	5	3	9	5	2	6	8	0	6
**105**	2	2	5	5	2	5	10	5	1
**110**	2	0	3	3	2	1	2	2	5
**115**	1	5	3	1	2	2	5	4	5
**120**	1	1	1	7	0	6	5	2	1
**125**	0	2	1	4	0	2	1	4	1
**130**	3	0	1	2	0	0	3	1	2
**135**	1	2	1	0	0	1	2	1	0
**140**	3	2	1	1	1	0	2	1	2
**145**	0	1	0	1	0	2	0	2	1
**150**	2	0	3	2	2	2	0	0	1
**155**	0	1	1	1	0	1	1	0	1
**160**	0	3	0	2	2	1	2	0	0
**165**	0	0	0	1	0	1	1	1	0
**170**	0	0	0	0	0	0	0	0	0
**175**	0	1	1	1	0	0	1	0	0
**180**	0	1	1	0	1	1	0	0	1
**185**	0	0	0	0	0	1	0	1	0
**190**	2	0	0	1	0	1	0	0	1
**195**	0	0	1	0	0	1	1	0	0
**200**	1	1	1	0	0	0	1	0	0

5d RPM									
	**CD63**			**CD81**			**CD9**		
**Size (nm)**	**A**	**B**	**C**	**A**	**B**	**C**	**A**	**B**	**C**
**50**	538	607	328	234	158	84	440	489	285
**55**	1147	1249	449	715	539	157	1064	1070	528
**60**	398	374	67	425	342	56	471	430	176
**65**	249	278	29	366	285	39	299	221	97
**70**	143	218	12	294	200	22	211	147	41
**75**	118	150	3	192	145	7	138	101	36
**80**	94	146	9	119	133	9	85	79	18

**85**	60	97	5	71	87	1	46	42	14
**90**	49	76	1	44	71	1	38	42	8
**95**	43	57	1	31	49	1	25	29	6
**100**	40	39	2	26	32	4	13	21	5
**105**	33	46	0	12	24	2	8	15	5
**110**	23	38	2	8	18	1	10	14	7
**115**	9	22	1	6	21	0	6	7	4
**120**	17	29	0	2	16	1	9	3	2
**125**	11	18	0	3	14	0	4	7	2
**130**	12	25	2	5	15	1	5	5	2
**135**	12	9	1	3	9	0	3	6	0
**140**	15	7	0	2	13	0	3	5	1
**145**	12	6	0	3	5	1	2	5	1
**150**	8	4	1	3	14	0	1	4	1
**155**	5	2	0	1	5	0	0	2	1
**160**	7	1	1	5	3	0	3	3	0
**165**	7	8	0	0	2	0	2	1	0
**170**	10	5	1	1	6	0	1	2	0
**175**	6	5	0	3	2	1	1	1	0
**180**	5	5	0	1	1	0	1	2	0
**185**	2	5	0	0	0	0	2	2	0
**190**	3	4	0	2	1	0	1	0	0
**195**	3	5	0	1	0	0	0	1	0
**200**	3	2	0	2	1	0	0	0	0

**10d 1*g***									
	**CD63**			**CD81**			**CD9**		
**Size (nm)**	**A**	**B**	**C**	**A**	**B**	**C**	**A**	**B**	**C**
**50**	359	281	244	81	169	60	274	382	325
**55**	531	564	378	121	510	83	498	887	561
**60**	60	228	55	37	294	26	129	395	163
**65**	21	154	27	22	230	18	52	243	83
**70**	10	124	12	13	128	6	40	150	41
**75**	15	98	14	9	99	3	15	110	26
**80**	3	60	6	4	78	1	15	90	24
**85**	4	50	6	8	66	1	12	67	14
**90**	2	33	5	4	45	1	9	53	8
**95**	4	32	2	2	36	0	5	39	9
**100**	3	21	2	3	22	0	5	27	4
**105**	2	19	1	1	26	1	3	24	3
**110**	2	15	3	1	23	2	0	14	2
**115**	0	5	0	2	23	1	0	11	2
**120**	1	7	0	1	13	0	0	11	1
**125**	0	4	0	0	5	0	1	10	2
**130**	1	5	1	0	10	0	1	2	1
**135**	0	2	1	0	6	0	1	4	0

**140**	1	3	0	0	6	1	0	3	0
**145**	0	4	1	2	5	1	0	5	0
**150**	0	4	0	1	0	1	1	2	0
**155**	0	2	1	0	4	0	0	3	2
**160**	0	3	0	0	7	0	0	0	2
**165**	0	1	0	0	1	0	0	2	0
**170**	0	1	0	1	1	0	0	1	0
**175**	0	2	0	0	1	0	0	2	0
**180**	0	3	0	0	1	0	0	0	1
**185**	0	0	0	0	2	0	0	1	0
**190**	0	1	0	0	1	1	0	0	0
**195**	1	1	1	0	1	0	0	0	1
**200**	0	1	0	0	0	0	0	1	0

**10d RPM**									
	**CD63**			**CD81**			**CD9**		
**Size (nm)**	**A**	**B**	**C**	**A**	**B**	**C**	**A**	**B**	**C**
**50**	425	445	447	127	182	96	374	399	378
**55**	586	815	684	211	454	265	653	856	742
**60**	91	302	106	79	204	68	195	357	195
**65**	46	186	42	43	181	26	102	217	104
**70**	27	140	23	28	108	12	58	131	62
**75**	12	103	19	12	74	16	47	94	38
**80**	8	100	10	12	75	4	22	92	16
**85**	7	76	9	11	59	1	16	46	12
**90**	3	40	4	4	35	1	20	48	10
**95**	6	42	4	4	30	1	8	33	8
**100**	4	31	1	3	21	2	7	26	3
**105**	3	28	6	3	24	2	6	18	6
**110**	3	16	5	1	9	1	3	14	4
**115**	1	13	1	2	10	1	5	9	4
**120**	1	13	2	1	6	2	3	9	2
**125**	4	4	0	0	5	1	3	10	2
**130**	2	9	3	3	3	1	1	9	0
**135**	0	4	1	1	8	0	0	7	2
**140**	0	5	1	1	0	0	0	3	1
**145**	0	8	0	0	4	0	0	4	0
**150**	1	1	2	0	3	0	1	2	1
**155**	1	4	1	0	1	0	0	1	1
**160**	2	2	1	3	2	0	0	0	2
**165**	0	3	1	1	3	0	1	2	0
**170**	0	0	0	2	2	0	1	2	2
**175**	1	2	0	0	1	0	2	2	0
**180**	0	0	0	0	0	0	0	1	0
**185**	1	1	0	0	0	0	0	1	1
**190**	0	1	0	0	2	0	1	1	0

**195**	1	0	0	0	0	0	0	0	0
**200**	0	0	0	0	0	0	1	0	0
